# Supplementary material for: The Potential of Using Generative AI/NLP to Identify and Analyse Critical Incidents in a Critical Incident Reporting System (CIRS): A Feasibility Case–Control Study
Source: Healthcare (Basel). 2024 Oct 2;12(19):1964. doi: 10.3390/healthcare12191964 (PMC11475821; doi:10.3390/healthcare12191964)
Supplement: Supplementary file 1 [file healthcare-12-01964-s001.zip › Supplementary/Example_review.pdf]

Report

Title: Disconnection between tube and connector
Responsible speciality. Anaesthesiology
Case description: Disconnection between the endotracheal tube and the connector often occurs with the tubes we use. Regular inspection of the connector is therefore essential. This was not necessary before: other production processes?
What was particularly good: *blank*
What was particularly unfavourable: *blank*
Own advice (take-home-messa... Use tubes with glued connector?! Report to company?
How often does an event of this kind occur? Once every week
Who reports: *blank*
Free text for Who reports *blank*
Your professional experience: *blank*

Review Human

No recommendation

Not really a CIRS case

Review AI/NLP

Frequent disconnection between tube and connector

Lack of regular testing of the connector

Potentially problematic production processes for the tubes used

Recommendation for the use of tubes with bonded connector

Consideration of a report to the manufacturer due to the repeated occurrence of this event

High frequency of this event (weekly)

For the full list of CIRS cases used in this study, please contact the corresponding author.

Report

Title:	Operating table blocked in head-down position during the spread phase of spinal anaesthesia
Speciality:	Anaesthesiology
Free text for responsible speciality	*blank*
Where did the event happen?	Hospital
Free text for Where did the event happen?	
Day of the reported event:	Weekday
What type of care:	Routine OP
ASA classification:	ASA I
Patient condition:	Pregnant woman for elective caesarean section
Important accompanying circumstances:	*blank*
Case description:	Spinal anaesthesia is administered without difficulty for elective caesarean section and a dose of bupivacaine is applied that is usual in the department. Because the spinal anaesthesia is inadequate and hesitant to spread, the patient is placed in a slight head-down position by tilting the operating table with the remote control. When the patient complains about a slight dizziness/dyspnoea, the table is intended to be brought back into a horizontal position to stop the spinal anaesthesia from rising any further. However, the table cannot be moved with the remote control. Even after plugging the table cable into the charging current, the table will not move. The table can only be moved again using the remote control after a certain charging time; the table cannot be moved manually, nor is there any manual tilting option.<br>The control panel on the column of the operating table does not work either.
What was particularly good:	The spinal anaesthesia, which was actually only partially sufficient despite prolonged head-down position only just up to the ribcage. Two unfavourable factors come together and make the whole thing turn out badly
What was particularly unfavourable:	- there is no manual tilting option for the operating table when the battery is unloaded<br>- the table also needs a certain amount of time on the load before it can be operated again.
Own advice	Discussion with theatre nursing staff: Table will otherwise be moved Charged at the weekend, presentation of medical technology, discussion: shorten charging intervals or leave permanently connected to the power supply
Who reports:	Physican
Free text for Who reports	*blank*
Your professional experience:	over 5 years

Review Human

Technical malfunction

No redundancy available for table operation, reliant on electricity

Various possible solutions: Technical change (expensive?), protocol/working procedure that ensures regular loading of the table

Also indispensable: Plan "B" for renewed malfunction (e.g. manual "repositioning" of patients - who, how, etc.).

Review AI/NLP

No manual tilting option for the operating table

Insufficient charging power supply to the operating table, which leads to delays in operability

Need for discussion with regard to improving the charging intervals or permanent power supply to the table

For the full list of CIRS cases used in this study, please contact the corresponding author.
